# Does BMI Have an Impact on Endometriosis Symptoms and Endometriosis Types According to the #ENZIAN Classification?

**DOI:** 10.3390/jcm14124040

**Published:** 2025-06-07

**Authors:** Elvin Piriyev, Clara Mennicken, Sven Schiermeier, Thomas Römer

**Affiliations:** 1 Chair of Gynecology and Obstetrics, University Witten-Herdecke, 58455 Witten, Germany; 2 Department of Obstetrics and Gynecology, Academic Hospital Cologne Weyertal, University of Cologne, 50931 Cologne, Germany; 3 Chair of Gynecology and Obstetrics, University of Cologne, 50923 Cologne, Germany

**Keywords:** endometriosis, BMI, #ENZIAN classification, symptoms, chronic pelvic pain, infertility

## Abstract

**Background/Objectives:** The relationship between body mass index (BMI) and endometriosis symptoms or lesion types remains unclear. This study investigates the association between BMI and symptom severity as well as the anatomical distribution of endometriosis using the #ENZIAN classification. **Methods**: A retrospective analysis was conducted on 219 patients with histologically confirmed endometriosis who underwent laparoscopic surgery at a tertiary endometriosis center in 2021. Preoperative symptom data were collected using standardized questionnaires. Patients were grouped by BMI categories based on WHO criteria. Endometriosis was classified intraoperatively using the #ENZIAN system. Statistical analyses included chi-square tests and one-way ANOVA. **Results**: Patients with low/normal BMI (<25 kg/m^2^, n = 150) reported significantly higher intensity of chronic pelvic pain (CPP) compared to those with overweight/obesity (≥25 kg/m^2^, n = 69; *p* = 0.0026). When stratified into four BMI groups, dyspareunia was significantly less frequent in obese patients (*p* = 0.0306), and high-intensity CPP was less common in both underweight and obese categories compared to normal-weight patients (*p* = 0.0069). Infertility rates increased significantly with higher BMI (*p* = 0.00001). No significant differences in the distribution of endometriosis lesions across #ENZIAN compartments were observed in relation to BMI. **Conclusions**: Our findings indicate that BMI does not significantly influence the anatomical distribution of endometriosis lesions as defined by the #ENZIAN classification, but it does correlate with some symptom intensity and infertility. These results suggest that while BMI may not predict disease localization, it plays a role in shaping the clinical phenotype of endometriosis.

## 1. Introduction

Endometriosis is one of the most common benign gynecological conditions, characterized by the ectopic implantation of endometrial tissue outside the uterus, typically accompanied by chronic inflammation. Despite extensive research, the pathogenesis of endometriosis remains largely unclear [1,2]. Its epidemiology is also not fully understood [3]; however, current data suggest that up to 10% of women of reproductive age may be affected [4].

The disease manifests with a wide range of symptoms, most commonly including dysmenorrhea, dyspareunia, dysuria, and dyschezia [5]. However, the symptom spectrum extends beyond these. Chronic pelvic pain (CPP), gastrointestinal complaints such as nausea, vomiting, diarrhea, constipation, and bloating, as well as urinary symptoms like increased frequency and cystitis, may also be present [6,7,8,9]. In cases of deep infiltrating endometriosis involving the bowel or bladder, catamenial bleeding may occur in stool or urine [10,11]. Moreover, endometriosis is a well-established cause of infertility [4].

Accurate classification of endometriosis is essential for evaluating disease severity, identifying lesion location, and guiding clinical management. The #ENZIAN classification system is widely accepted and provides a detailed assessment of peritoneal, ovarian, and deep endometriosis across various anatomical compartments, as well as tubo-ovarian adhesions, based on surgical findings [12]. Compared to the revised American Society for Reproductive Medicine (rASRM) classification—which primarily focuses on peritoneal endometriosis, adhesions, and endometriomas [13]—the #ENZIAN system offers a more comprehensive description, particularly for deep infiltrating endometriosis.

The relationship between body mass index (BMI) and endometriosis remains controversial. While some studies report no significant association—or only a marginal trend—between low BMI and the presence of endometriosis at diagnosis, others have demonstrated a significant negative correlation [14]. Epidemiologic studies also examining BMI and endometriosis have generally indicated an inverse correlation between the two (i.e., higher BMI is associated with a lower risk of endometriosis) [15]. In particular, endometriosis appears to be more prevalent among lean or underweight women and less common in women who are overweight or obese relative to those of normal BMI [15,16]. For example, a meta-analysis reported approximately a 33% decrease in endometriosis risk for every 5 kg/m^2^ increase in BMI and noted that obese women had significantly lower odds of endometriosis compared to normal-weight controls. This evidence collectively suggests a negative association between adiposity and endometriosis prevalence across the spectrum of BMI categories [15].

A growing body of evidence suggests that body composition may influence endometriosis pathophysiology and presentation. Adipose tissue is not merely an inert energy store but a highly active endocrine organ: it produces adipokines (e.g., leptin, adiponectin, resistin) and inflammatory cytokines that regulate immune responses, angiogenesis, and metabolism [17]. These mediators modulate estrogen signaling (via aromatization in fat) and inflammation, processes central to endometriotic lesion growth. Notably, women with endometriosis often have lower BMI and altered fat distribution (more peripheral subcutaneous fat) compared to unaffected women [18]. Obesity creates a pro-inflammatory milieu, while leanness is associated with shifts in immune cell polarization (e.g., M2 macrophages) [18]. Since chronic inflammation and estrogen dependence are hallmarks of endometriosis, differences in adiposity could plausibly affect lesion characteristics and symptoms [17,18]. This provides a biological and clinical rationale to examine whether BMI correlates with symptom severity or endometriosis subtype (e.g., deep infiltrating lesions) under the #ENZIAN classification. In other reproductive disorders (e.g., PCOS, infertility) adiposity is known to influence hormonal and immune factors; similar links may exist in endometriosis.

The aim of the present study was to evaluate the impact of BMI on endometriosis. The primary objective was to assess the relationship between BMI and endometriosis symptoms. The secondary objective was to examine the association between BMI and different types of endometriosis as classified by the #ENZIAN score.

## 2. Materials and Methods

This retrospective study analyzed endometriosis cases treated at Academic Hospital Weyertal, a Level III Endometriosis Center of Excellence. All patients presenting to the endometriosis consultation at our center are routinely asked to complete a standardized, self-administered questionnaire, which includes a subjective assessment of symptoms. Key pain symptoms were rated individually on a 0–10 visual analog scale (VAS), where 0 = no pain and 10 = worst imaginable pain. The questionnaire explicitly asked about dysmenorrhea (menstrual pain), deep dyspareunia (painful intercourse), non-cyclic chronic pelvic pain, dyschezia (pain with bowel movements), dysuria (urinary pain) and other endometriosis-related symptoms We chose the VAS/NRS format because it is the most commonly used and validated method for endometriosis pain measurement [19]. Numerical values (0–10) were recorded for analysis. Demographic and gynecological data were also collected. BMI was calculated from measured height and weight at the preoperative visit.

We reviewed medical records from patients treated in 2021. Only those who underwent laparoscopic excision of endometriosis were considered for inclusion. The inclusion criteria were as follows: (1) a fully completed preoperative questionnaire, (2) endometriosis as the main diagnosis, (3) histological confirmation of endometriosis, and (4) complete BMI data. After excluding duplicate entries, a total of 219 patients were included in the final analysis. A diagnosis of endometriosis was confirmed only when endometrial-type glands and stroma were identified outside the uterus. In other words, lesions had to contain at least endometrial glands and/or stroma to meet diagnostic criteria. Cases without histological confirmation were excluded from the analysis. This ensured that only women with pathologically verified endometriosis were included.

Intraoperative classification of endometriosis was performed according to the #ENZIAN classification system [12]. This classification provides a detailed mapping of endometriosis lesions by site and depth. It includes the following components:

- P: Peritoneum
- O: Ovaries
- T: Adhesions of the tubo-ovarian unit
- Deep infiltrating endometriosis (DIE), which is further subdivided into:
  ◦ A: Vagina and rectovaginal space
  ◦ B: Uterosacral and cardinal ligaments, pelvic sidewall
  ◦ C: Rectum
- F: Other locations, including adenomyosis (FA), urinary bladder (FB), ureters (FU), and bowel (FI) [12].

Body mass index (BMI) was calculated as weight in kilograms divided by height in meters squared (kg/m^2^) and categorized according to World Health Organization (WHO) criteria [20]:

- BMI < 18.5: Underweight
- BMI 18.5–24.9: Normal weight
- BMI ≥ 25.0: Overweight
- BMI ≥ 30.0: Obesity

Three statistical analyses were conducted:

1. Patients were initially categorized into two groups: Group 1 included those with underweight or normal BMI (BMI < 25), and Group 2 included those who were overweight or obese (BMI ≥ 25).
2. For a more nuanced analysis, patients were further stratified into four subgroups:
  ◦ Group 1: BMI < 18.5
  ◦ Group 2: BMI 18.5–24.9
  ◦ Group 3: BMI 25.0–29.9
  ◦ Group 4: BMI ≥ 30.0
3. A reverse analysis was performed by dividing patients based on the type of endometriosis: those with DIE (compartments A, B, C) versus those with peritoneal or ovarian endometriosis without DIE (compartments P and O). A subanalysis also compared patients with rectal involvement (compartment C) to those without DIE.
  To further explore the interaction between BMI and endometriosis phenotype on symptom presentation, patients were additionally subdivided based on BMI into two subgroups: BMI < 25 kg/m^2^ and BMI ≥ 25 kg/m^2^. Symptom frequencies were compared across BMI subgroups within each phenotype group to identify potential BMI-related differences in clinical presentation.

To our knowledge, this is the first study investigating the relationship between BMI and both the symptom profile and classification of endometriosis using the #ENZIAN system.

Statistical analysis was performed using chi-square tests for contingency tables (up to 5 × 5), with a significance threshold of *p* < 0.05. Descriptive statistics were calculated, including means and standard deviations. One-way ANOVA with Tukey HSD post hoc analysis was used to compare means across groups. Results are presented as mean ± standard deviation (SD). Odds ratios (OR) and adjusted odds ratios (aOR) with 95% confidence intervals (CI) were calculated using logistic regression models to assess the association between BMI categories and the presence of specific endometriosis symptoms, adjusting for potential confounders including age.

## 3. Results

A total of 219 patients with histologically confirmed endometriosis were included in the analysis. Based on BMI, patients were categorized into two main groups: Group 1 (low/normal BMI, <25 kg/m^2^; n = 150) and Group 2 (overweight/obese, ≥25 kg/m^2^; n = 69). The mean BMI differed significantly between the groups (21.43 ± 2.11 vs. 30.03 ± 4.33 kg/m^2^; *p* = 0.0001), while the mean age was comparable (33.51 ± 6.85 vs. 33.60 ± 7.42 years; *p* = 0.9356).

### 3.1. First Analysis (Table 1 and Table 2)

The overall prevalence of symptoms such as dysmenorrhea, dyspareunia, dyschezia, and chronic pelvic pain (CPP) was high in both BMI groups. While the frequency of these symptoms did not differ significantly between groups, patients with low/normal BMI reported significantly higher CPP intensity (*p* = 0.0026 for VAS > 5; *p* = 0.0141 for VAS < 5), indicating a greater pain burden in this subgroup. Other symptoms, including abnormal uterine bleeding (AUB), nausea/vomiting, and gastrointestinal complaints, did not show statistically significant differences between the groups (Table 1).

**Table 1:** Comparison of symptoms between patients with low/normal BMI and those with overweight/obesity (First Analysis).

	Group 1	Group 2	*p*-Value
N of patients	150	69	
Age mean ± SD	33.51 ± 6.85	33.60 ± 7.42	0.9356
BMI mean ± SD kg/m^2^	21.43 ± 2.11	30.03 ± 4.33	**0.0001**
Dysmenorrhea	147 (98%)	66 (95.6%)	0.3227
>5 VAS	137 (91.3%)	61 (88.4%)	0.4942
<5 VAS	10 (6.7%)	5 (7.2%)	0.8746
Dyspareunia	110 (73.3%)	44 (63.8%)	0.1500
>5 VAS	37 (24.7%)	18 (26.1%)	0.8218
<5 VAS	73 (48.7%)	26 (37.7%)	0.1291
Dyschezia	103 (68.7%)	38 (55.1%)	0.0509
>5 VAS	31 (20.7%)	10 (14.5%)	0.2765
<5 VAS	72 (48%)	28 (40.6%)	0.3057
Dysuria	68 (45.3%)	24 (34.8%)	0.1416
>5 VAS	12 (8%)	3 (4.3%)	0.3202
<5 VAS	56 (37.3%)	21 (30.4%)	0.3205
Hematochezia	29 (19.3%)	14 (20.3%)	0.8685
Hematuria	30 (20%)	8 (11.6%)	0.1270
CPP	135 (90%)	59 (85.5%)	0.3314
>5 VAS	85 (56.7%)	24 (34.8%)	**0.0026**
<5 VAS	50 (33.3%)	35 (50.7%)	**0.0141**
Monthly need for analgesia (days) mean ± SD	5.04± 5.51	5.92± 5.18	0.2453
Nausea/Vomiting	80 (53.3%)	45 (65.2%)	0.0988
Diarrhea/Obstipation/Bloating	138 (92%)	65 (94.2%)	0.5606
AUB	96 (64%)	53 (76.8%)	0.0589
Infertility	32 (21.3%)	21 (30.4%)	0.1440

CPP—chronic pelvic pain, VAS—visual analog scale, AUB—abnormal uterine bleeding.

Evaluation of endometriosis localization based on the #ENZIAN classification showed no significant differences between BMI groups regarding the involvement of compartments P, O, T, A, B, or C. The prevalence of adenomyosis (FA) was slightly higher in the overweight/obese group compared to the low/normal BMI group (91.3% vs. 84.7%); however, this difference was not statistically significant (*p* = 0.1782) (Table 2).

**Table 2:** Comparison of endometriosis lesions according to the #ENZIAN classification between patients with low/normal BMI and those with overweight/obesity (First Analysis).

#ENZIAN	Group 1 (150 P)	Group 2 (69 P)	*p*-Value
P	110 (73.3%)	53 (76.8%)	0.5836
O	21 (14%)	6 (8.7%)	0.2673
bilateral	6 (4%)	2 (2.9%)	0.6864
unilateral	15 (10%)	4 (5.8%)	0.3046
T	28 (18.7%)	13 (18.8%)	0.9755
bilateral	8 (5.3%)	5 (7.2%)	0.5778
unilateral	20 (13.3%)	8 (11.6%)	0.7203
A	26 (17.3%)	10 (14.5%)	0.5982
B	34 (22.7%)	17 (24.6%)	0.7485
bilateral	16 (10.7%)	6 (8.7%)	0.6521
unilateral	18 (12%)	11 (15.9%)	0.4239
C	14 (9.3%)	7 (10.1%)	0.8497
FA	127 (84.7%)	63 (91.3%)	0.1782

### 3.2. Extended Analysis Across Four BMI Categories (Second Analysis, Table 3 and Table 4)

When stratifying patients into four BMI categories (<18.5, 18.5–24.9, 25.0–29.9, and ≥30.0 kg/m^2^), no significant differences were observed in age or general symptom prevalence. However, a statistically significant decrease in dyspareunia was noted in the obese group (46.2%, *p* = 0.0306), as well as a lower proportion of patients reporting high-intensity CPP in both the underweight and obese categories compared to the normal BMI group (*p* = 0.0069). Additionally, infertility showed a clear trend of increasing prevalence with rising BMI, reaching 38.5% in patients with BMI ≥ 30 kg/m^2^ (*p* = 0.00001) (Table 3).

**Table 3:** Comparison of endometriosis-related symptoms in patients according to BMI categories (Second Analysis).

BMI kg/m^2^	<18.5	18.5–24.9	25.0–29.9	≥30.0	*p*-Value
N of patients	12	138	43	26	
Age mean ± SD	33.58 ± 7.57	33.32 ± 7.27	32.67 ± 7.08	35.12 ± 7.84	0.5844
BMI mean ± SD kg/m^2^	17.52 ± 0.94	21.77 ± 1.81	27.48 ± 1.40	34.26 ± 4.23	**0.0002**
Dysmenorrhea	12 (100%)	135 (97.8%)	41 (95.3%)	25 (96.2%)	0.6476
>5 VAS	9 (75%)	128 (92.7%)	38 (88.4%)	23 (88.5%)	0.2139
<5 VAS	3 (25%)	7 (5.1%)	3 (6.9%)	2 (7.7%)	0.0748
Dyspareunia	10 (83.3%)	100 (72.5%)	32 (74.4%)	12 (46.2%)	**0.0306**
>5 VAS	6 (50%)	31 (22.5%)	14 (32.6%)	4 (15.4%)	0.0705
<5 VAS	4 (33.3%)	73 (52.9%)	18 (41.9%)	8 (30.8%)	0.1100
Dyschezia	7 (58.3%)	96 (69.6%)	23 (53.5%)	15 (57.7%)	0.2086
>5 VAS	3 (25%)	28 (20.3%)	4 (9.3%)	6 (23.1%)	0.3386
<5 VAS	4 (33.3%)	68 (49.3%)	9 (20.9%)	9 (34.6%)	**0.0081**
Dysuria	4 (33.3%)	64 (46.4%)	15 (34.9%)	9 (34.6%)	0.4023
>5 VAS	0 (0%)	12 (8.7%)	2 (4.6%)	1 (3.8%)	0.7270
<5 VAS	4 (33.3%)	52 (37.7%)	13 (30.2%)	8 (30.8%)	0.7818
Hematochezia	1 (8.3%)	28 (20.3%)	8 (18.6%)	6 (20.1%)	0.7451
Hematuria	2 (16.7%)	28 (20.3%)	5 (11.6%)	3 (11.5%)	0.4881
CPP	10 (83.3%)	125 (90.6%)	37 (86%)	22 (84.6%)	0.6709
>5 VAS	4 (33.3%)	81 (58.7%)	14 (32.6%)	10 (38.5%)	**0.0069**
<5 VAS	6 (50%)	44 (31.9%)	23 (53.5%)	12 (46.2%)	**0.0478**
Monthly need for analgesia (days) mean ± SD	4.08 ± 3.38	5.12 ± 5.66	5.57 ± 4.42	6.52 ± 6.29	0.5293
Nausea/Vomiting	7 (58.3%)	73 (52.9%)	28 (65.1%)	17 (65.4%)	0.4140
Diarrhea/Obstipation/Bloating	11 (91.7%)	127 (92%)	41 (95.3%)	24 (92.3%)	0.9050
AUB	7 (58.3%)	89 (64.5%)	33 (76.7%)	20 (76.9%)	0.2885
Infertility	2 (16.7%)	30 (21.7%)	11 (25.6%)	10 (38.5%)	**0.00001**

CPP—chronic pelvic pain, VAS—visual analogue scale, AUB—abnormal uterine bleeding.

The logistic regression analysis revealed notable associations between BMI and specific endometriosis symptoms (infertility and CPP) (Table 4). Patients with obesity (BMI ≥ 30 kg/m^2^) had more than twice the odds of infertility compared to non-obese patients, a trend approaching statistical significance after age adjustment (age-adjusted OR = 2.30; 95% CI: 0.97–5.47; *p* = 0.060). Furthermore, patients with low or normal BMI (<25 kg/m^2^) showed significantly higher odds of experiencing high-intensity chronic pelvic pain (CPP, VAS > 5) compared to overweight or obese patients (age-adjusted OR = 2.22; 95% CI: 1.17–4.28; *p* = 0.015), underscoring a meaningful link between lower BMI and severe pelvic pain.

**Table 4:** Logistic Regression Analysis for BMI and Endometriosis Symptoms.

Symptom	BMI Comparison	Odds Ratio (95% CI)	*p*-Value
Infertility	Obese (≥30 kg/m^2^) vs. non-obese (<30 kg/m^2^)	2.18 (0.92–5.15)	0.071
Infertility (age-adjusted)	Obese (≥30 kg/m^2^) vs. non-obese (<30 kg/m^2^)	2.30 (0.97–5.47)	0.060
High-intensity CPP (VAS > 5)	Low/Normal (<25 kg/m^2^) vs. Overweight/Obese (≥25 kg/m^2^)	2.45 (1.36–4.43)	0.006
High-intensity CPP (VAS > 5, age-adjusted)	Low/Normal (<25 kg/m^2^) vs. Overweight/Obese (≥25 kg/m^2^)	2.22 (1.17–4.28)	0.015

CPP—chronic pelvic pain.

Analysis of lesion distribution using the #ENZIAN classification across the four BMI categories again demonstrated no statistically significant differences in the involvement of pelvic compartments (P, O, T, A, B, C). Adenomyosis is universally present in underweight patients (100%); however, this difference was not statistically significant (*p* = 0.2139) (Table 5).

### 3.3. Reverse Analysis by Endometriosis Type

Patients were regrouped based on lesion characteristics into those with deep infiltrating endometriosis (DIE, n = 66), patients without DIE (n = 153), and those with compartment C involvement (n = 21). No significant differences were found in age or BMI between these subgroups. The presence of DIE or compartment C involvement was not associated with higher or lower BMI (Table 6).

When stratifying patients by lesion phenotype and BMI, we observed notable differences in symptom presentation (Table 7). Among patients with deep infiltrating endometriosis (DIE), those with a BMI ≥ 25 kg/m^2^ reported significantly lower rates of dyschezia (45% vs. 84.8%, *p* = 0.0056) and no cases of dysuria (*p* = 0.0047). Additionally, chronic pelvic pain with VAS > 5 was less frequent in the higher BMI subgroup without DIE (*p* = 0.0144), suggesting BMI may influence symptom perception or manifestation.

We performed a post hoc power analysis for the primary pain outcome (chronic pelvic pain with VAS > 5) comparing Group 1 vs. Group 2. Using α = 0.05 and a target power of 80%, the analysis revealed an achieved power of 86.2%, indicating that our sample was sufficiently powered.

## 4. Discussion

Obesity and endometriosis are both common conditions characterized by systemic inflammation. The finding of an inverse association between these two conditions is intriguing but not yet fully understood [21,22]. Several studies have reported a relationship between BMI and endometriosis, suggesting a higher risk of endometriosis in patients with lower BMI [23]. Additionally, other research has indicated that DIE is more frequently associated with a low BMI [14]. These studies typically compared patients with endometriosis to control groups without the disease. In contrast, our analysis focused on evaluating the impact of BMI on symptom severity and lesion distribution according to the #ENZIAN classification in histologically confirmed cases of endometriosis. This retrospective analysis is, to our knowledge, the first to systematically evaluate the association between body mass index (BMI) and endometriosis phenotypes based on the anatomically detailed #ENZIAN classification system. Our results demonstrate that BMI does not significantly influence the anatomical distribution of endometriosis lesions across pelvic compartments (P, O, T, A, B, C, FA) or the prevalence of DIE in general. However, we observed notable differences in symptom intensity and infertility rates among BMI categories, highlighting a potential role of BMI in shaping the clinical presentation rather than the anatomical manifestation of the disease.

Specifically, we observed a significantly higher intensity of chronic pelvic pain (CPP) in patients with normal BMI compared to underweight and obese patients. This finding challenges assumptions that higher adiposity uniformly amplifies pain in endometriosis and may reflect differences in pain perception, reporting behaviors, or neuroinflammatory pathways between BMI groups [24,25]. Interestingly, the monthly need for analgesia was similar in the groups. However, many factors influence analgesic use, including individual pain tolerance, coping strategies, access to care, and personal preferences or concerns about medication. For example, some patients with high pain levels may avoid taking more painkillers due to fear of side effects or dependence, while others with moderate pain might take regular medication as a precaution. Indeed, prior research in chronic pain populations has shown that higher analgesic consumption does not necessarily equate to higher pain intensity—and in some cases, an inverse relationship is observed [26]. This underscores that analgesic usage is an imperfect proxy for pain severity. After adjusting for age, patients with a BMI < 25 kg/m^2^ had 2.22 times higher odds of experiencing high-intensity chronic pelvic pain compared to those with a BMI ≥ 25 kg/m^2^ (unadjusted OR = 2.45, 95% CI: 1.36–4.43; age-adjusted OR = 2.22, 95% CI: 1.17–4.28), highlighting a significant association between lower BMI and severe CPP (Table 4). Interestingly, dyspareunia was significantly less prevalent in obese women, suggesting that increased BMI may be associated with altered pelvic biomechanics or variations in sexual activity; however, psychological and hormonal factors might also contribute [27]. Furthermore, infertility showed a marked increase with rising BMI, with the highest prevalence in the obese group (38.5%), which is consistent with the established literature linking obesity to subfertility due to both ovulatory dysfunction and endometrial receptivity alterations [28]. In our cohort, patients with obesity (BMI ≥ 30 kg/m^2^) had more than twice the odds of infertility compared to non-obese patients (unadjusted OR = 2.18, 95% CI: 0.92–5.15; age-adjusted OR = 2.30, 95% CI: 0.97–5.47), suggesting a clinically relevant trend toward increased infertility risk among women with obesity (Table 4). Our analysis found no significant correlation between infertility and the #ENZIAN scores (Table 7).

Endometriosis pathophysiology is profoundly modulated by body fat–derived signals. Adipose tissue in obesity secretes pro-inflammatory cytokines and adipokines that amplify lesion inflammation and nociception. For example, peritoneal leptin—elevated in obese patients—correlates with disease stage and chronic pelvic pain [29]. By contrast, adiponectin (an anti-inflammatory, anti-angiogenic adipokine) is suppressed in endometriosis, so excess fat removes a protective brake on inflammation [22]. Obesity also enriches other proinflammatory adipokines (resistin, visfatin) in endometriotic lesions, driving local cytokine release [30]. These mediators sensitize pelvic nociceptors (via NGF, cytokines, etc.) and heighten pain signaling. In lean women, the immune milieu differs; low BMI is associated with an M2 macrophage–skewed profile that promotes angiogenesis and ectopic lesion survival [22]. This may paradoxically enhance nerve ingrowth into lesions and explain why lean patients often report worse pain despite less adiposity.

Endocrine effects also diverge by BMI. High adiposity raises aromatase activity and systemic estrogens, and obesity-induced insulin resistance elevates IGF-1 and adrenal androgens, which support ectopic cell survival while perturbing the hypothalamic–pituitary–ovarian axis [22]. Obesity thus often causes anovulation and luteal dysfunction, reducing menstrual reflux yet impairing fertility [22]. In sum, an obese milieu drives chronic inflammation and hormonal dysregulation that preserve lesions but compromise ovulation and endometrial receptivity, whereas low-BMI women with endometriosis experience an immune/endocrine state favoring lesion innervation and sensitization of pain pathways.

In this cohort, BMI modulated symptom profiles differently between patients with and without DIE (Table 6). Lean patients (BMI < 25) with DIE reported significantly more dyschezia, whereas overweight DIE patients (BMI ≥ 25) reported no dysuria. Chronic pelvic pain was also more severe in lower-BMI patients regardless of DIE status. These findings imply that lower adiposity amplifies pelvic pain and bowel symptoms, consistent with evidence linking low BMI to more extensive DIE [14]. Posterior DIE implants strongly correlate with dyschezia severity, suggesting that prevalent posterior lesions in lean women drive painful defecation [31]. Adipose-derived hormones and cytokines (e.g., leptin) promote systemic inflammation and angiogenesis in endometriosis. Obesity may thus alter lesion activity or sensory thresholds [31]. Conversely, low BMI might intensify neurogenic hyperalgesia. Overall, the interplay of inflammatory, neurological, and hormonal/metabolic factors appears to shape these BMI-specific symptom patterns [31,32]

The prevalence of adenomyosis (FA) in our study was notably high across all BMI categories, in contrast to previous studies that reported a positive correlation between higher BMI and the presence of adenomyosis [33,34]. This discrepancy may be attributed to improved recognition of adenomyosis and advances in imaging techniques in recent years, which have likely enhanced detection rates compared to the period when the cited studies were conducted. These factors may help explain the uniformly high prevalence of adenomyosis observed across all groups in our analysis. Notably, no significant differences in lesion (P, O, T, A, B, C, FA) distribution were observed between BMI groups, even when patients were categorized according to DIE versus non-DIE phenotypes, or by rectal involvement (compartment C). These results are consistent with the findings of Chapron et al., who reported no significant difference in BMI between patients with deep infiltrating endometriosis (DIE) and those with superficial disease [35]. Our data, derived from a cohort with histologically confirmed disease and standardized #ENZIAN mapping, suggest that BMI may not be a strong determinant of lesion depth or localization.

## 5. Clinical Applications and Future Directions

Our findings suggest that BMI-related factors could have practical implications in endometriosis management. For instance, stratifying patients by BMI may help tailor therapy: obese women (with higher inflammation and estrogen levels) might benefit from adjunct anti-inflammatory or metabolic interventions, whereas underweight women may require different pain management strategies. Surgeons could also consider BMI as part of preoperative counseling: both obesity and certain #ENZIAN lesion patterns (e.g., A3, B3, C1) are associated with higher complication rates, so knowing a patient’s BMI in advance might refine risk assessment. In addition, if higher adiposity is linked to more extensive pelvic involvement, medical treatment (e.g., aromatase inhibitors, hormonal suppression) might be optimized by weight or fat distribution. Conversely, recognizing a lean phenotype (often seen in endometriosis) could prompt the evaluation of metabolic factors that might influence recurrence risk. Overall, incorporating BMI into clinical decision-making (alongside #ENZIAN staging) could improve personalized care for endometriosis patients.

Future research should build on these observations. In particular, we recommend:

- Metabolic and hormonal profiling. Future studies should collect data on insulin resistance, lipid profiles, and circulating adipokines (leptin, adiponectin, inflammatory cytokines) in endometriosis patients. Correlating these markers with BMI and #ENZIAN stage may uncover mechanistic links between adiposity and lesion behavior.
- Prospective validation. Large, prospective cohorts should confirm whether BMI predicts disease progression or treatment response. For example, tracking symptoms and #ENZIAN scores over time in patients with different BMIs could test causality. Randomized trials of weight-loss or metabolic treatments in high-BMI endometriosis patients may also be warranted.
- Integration with biomarker discovery. Combining #ENZIAN staging with systemic biomarkers (e.g., serum cytokines, microRNAs) could improve diagnostic models. For instance, a composite score incorporating BMI and inflammatory markers might enhance early detection or recurrence risk stratification.
- Multidisciplinary management. Given the links between metabolism and gynecologic health, interdisciplinary approaches (gynecology, endocrinology, nutrition) should be explored. Trials of lifestyle interventions (diet, exercise) on endometriosis outcomes by BMI subgroup could inform comprehensive care.

Together, these directions aim to integrate metabolic and anthropometric insights into endometriosis research and care. In summary, understanding how adiposity interfaces with endometriosis can refine symptom stratification and guide both surgical and medical decision-making, ultimately leading to more personalized treatment strategies.

## 6. Conclusions

In conclusion, our findings indicate that BMI does not significantly influence the anatomical distribution of endometriosis lesions as defined by the #ENZIAN classification, but it does correlate with some symptom intensity and infertility. These results suggest that while BMI may not predict disease localization, it plays a role in shaping the clinical phenotype of endometriosis. Future prospective studies incorporating hormonal, metabolic, and imaging data could further elucidate the mechanistic pathways linking adiposity and endometriosis expression.

## 7. Strengths and Limitations

One of the strengths of this study is the rigorous application of the #ENZIAN classification, which provides a more nuanced assessment of lesion topography than the traditional rASRM staging. Additionally, by stratifying BMI both dichotomously and across WHO-defined categories, we captured trends that might have been missed in simpler binary analyses. The use of standardized preoperative questionnaires allowed for a homogeneous analysis. Furthermore, histological confirmation strengthens diagnostic accuracy.

However, the study has limitations. First, its retrospective design may introduce selection and reporting biases. However, we believe that even a prospective study would likely yield similar results. This is because, despite the retrospective nature, we used prospectively collected data, as it is our routine practice to systematically document all surgical findings using the #ENZIAN classification. Second, BMI, while widely used, is an imperfect proxy for body composition and does not account for fat distribution, which may have differential effects on hormonal and inflammatory profiles. Parameters such as fasting glucose, insulin resistance markers, sex-steroid levels, and waist-to-hip ratio would help disentangle the contributions of endocrine and metabolic factors from BMI per se. However, these assessments are not part of our routine preoperative evaluation, and the retrospective design precluded their inclusion. Future prospective studies should incorporate comprehensive metabolic and body composition profiling to clarify these mechanistic pathways. Third, although the preoperative symptom questionnaire was standardized, pain perception is inherently subjective and influenced by psychosocial factors not captured in our analysis. Fourth, we did not systematically assess coexisting chronic pain conditions such as fibromyalgia, interstitial cystitis, or irritable bowel syndrome, which could have influenced pain perception independently of BMI. However, as this limitation applies equally to all BMI groups, the potential bias is likely non-differential. Finally, the sample sizes in certain BMI subgroups—particularly the underweight and obese categories—were small, limiting the statistical power for some comparisons and requiring cautious interpretation of subgroup analyses.

## Figures and Tables

**Table 5:** Comparison of endometriosis lesion distribution according to the #ENZIAN classification based on BMI categories (Second Analysis).

Group	<18.5	18.5–24.9	25.0–29.9	≥30.0	*p*-Value
N of patients	12	138	43	26	
P	8 (66.7%)	102 (73.9%)	35 (81.4%)	18 (69.2%)	0.6009
O	1 (8.3%)	20 (14.5%)	2 (4.6%)	4 (15.4%)	0.3414
bilateral	0 (0%)	6 (4.3%)	1 (2.3%)	1 (3.8%)	0.8498
unilateral	1 (8.3%)	14 (10.1%)	1 (2.3%)	3 (11.5%)	0.4177
T	3 (25%)	25 (18.1%)	6 (13.9%)	7 (26.9%)	0.5446
bilateral	1 (8.3%)	7 (5.1%)	1 (2.3%)	4 (15.4%)	0.1405
unilateral	2 (16.7%)	8 (5.8%)	5 (11.6%)	3 (11.5%)	0.3546
A	2 (16.7%)	24 (17.4%)	6 (13.9%)	4 (15.4%)	0.9588
B	1 (8.3%)	33 (23.9%)	10 (23.3%)	7 (26.9%)	0.6314
bilateral	1 (8.3%)	15 (10.9%)	3 (6.9%)	3 (11.5%)	0.8837
unilateral	0 (0%)	18 (54.5%)	7 (16.3%)	4 (15.4%)	0.8605
C	0 (0%)	14 (10.1%)	3 (6.9%)	4 (15.4%)	0.7270
FA	12 (100%)	115 (83.3%)	41 (95.3%)	22 (84.6%)	0.2139

**Table 6:** Comparison of Age and BMI According to Endometriosis Type (Reverse Analysis).

Group	Patients Without DIE (P, O, T, FA)	Patients with DIE (A, B, C ± Others)	Patients with Compartment C Involvement
N	153	66	21
Age (mean ± SD)	32.97 ± 7.47	34.29 ± 6.72	33.05 ± 3.93
*p*-value of age		0.2179 *	0.9617 **
BMI (mean ± SD)	24.20 ± 5.00	23.93 ± 5.03	24.54 ± 5.74
*p*-value of BMI		0.7147 *	0.7745 **

* The *p*-value reflects the statistical comparison between Group 1 and Group 2. ** The *p*-value reflects the statistical comparison between Group 1 and Group 3.

**Table 7:** Comparison of Symptom Profiles by BMI in Patients With and Without Deep Infiltrating Endometriosis According to the #ENZIAN Classification.

	Patients Without DIE (P, O, T, FA)		Patients with DIE (A, B, C ± Others)		*p*-Value
**BMI kg/m** **^2^**	**<25**	≥25	<25	≥25	
N of patients	104	49	46	20	
Age mean ± SD	32.72 ± 7.37	33.50 ± 7.75	34.34 ± 6.80	33.85 ± 6.72	0.5648
BMI mean ± SD kg/m^2^	21.54 ± 2.12	29.98 ± 4.48	21.18 ± 2.11	30.17 ± 4.03	**0** **.0016**
Dysmenorrhea	102 (98.1%)	47 (95.9%)	45 (97.8%)	19 (95%)	0.7939
>5 VAS	94 (90.3%)	46 (93.8%)	43 (93.5%)	15 (75%)	0.0836
<5 VAS	8 (7.7%)	1 (2.1%)	2 (4.3%)	4 (20%)	0.0511
Dyspareunia	82 (78.8%)	33 (67.3%)	28 (60.9%)	11 (55%)	**0.** **0450**
>5 VAS	26 (25%)	14 (28.6%)	11 (23.9%)	4 (20%)	0.8905
<5 VAS	56 (53.8%)	19 (38.8%)	17 (36.9%)	7 (35%)	0.1088
Dyschezia	64 (61.5%)	29 (59.2%)	39 (84.8%)	9 (45%)	**0** **.0056**
>5 VAS	19 (18.3%)	8 (16.3%)	12 (26.1%)	2 (10%)	0.4172
<5 VAS	45 (43.3%)	21 (42.8%)	27 (58.7%)	7 (35%)	0.2157
Dysuria	48 (46.1%)	24 (48.9%)	20 (43.5%)	0	**0.** **0047**
>5 VAS	7 (6.7%)	3 (6.1%)	5 (10.9%)	0	0.7597
<5 VAS	41 (39.4%)	21 (42.8%)	15 (32.6%)	0	0.0174
Hematochezia	19 (18.3%)	12 (24.5%)	10 (21.7%)	2 (10%)	0.5397
Hematuria	20 (19.2%)	7 (14.3%)	10 (21.7%)	1 (5%)	0.3445
CPP	95 (91.3%)	43 (87.7%)	40 (86.9%)	16 (80%)	0.4944
>5 VAS	62 (59.6%)	16 (32.6%)	23 (50%)	8 (40%)	**0** **.0144**
<5 VAS	33 (31.7%)	27 (55.1%)	17 (36.9%)	8 (40%)	0.0514
Monthly need for analgesia (days) mean ± SD	5.03 ± 5.75	6.28 ± 5.40	5.27 ± 4.98	5.05 ± 4.59	0.5581
Nausea/Vomiting	52 (50%)	33 (67.3%)	28 (60.8%)	12 (60%)	0.2056
Diarrhea/Obstipation/Bloating	93 (89.4%)	47 (98.9%)	45 (97.8%)	18 (90%)	0.2215
AUB	67 (64.4%)	39 (79.6%)	29 (63.1%)	14 (70%)	0.2410
Infertility	21 (20.2%)	14 (28.6%)	11 (23.9%)	7 (35%)	0.4411

CPP—chronic pelvic pain, VAS—visual analogue scale, AUB—abnormal uterine bleeding.

## Data Availability

The raw data supporting the conclusions of this article will be made available by the authors on request.
